# Phytochemical analysis of medicinal plants of Nepal and their antibacterial and antibiofilm activities against uropathogenic *Escherichia coli*

**DOI:** 10.1186/s12906-021-03293-3

**Published:** 2021-04-09

**Authors:** Sudip Bhandari, Karan Khadayat, Sami Poudel, Sunil Shrestha, Raju Shrestha, Poonam Devkota, Santosh Khanal, Bishnu P. Marasini

**Affiliations:** 1 Department of Biotechnology, National College, Tribhuvan University, Naya Bazar, Kathmandu, Nepal; 2 Department of Microbiology, National College, Tribhuvan University, Naya Bazar, Kathmandu, Nepal

**Keywords:** Medicinal plants, Biofilm, Antibacterial activity, Antibiofilm activity

## Abstract

**Background:**

A biofilm is an extracellular polymeric substance (EPS) composed of polysaccharides, proteins, nucleic acids, and lipids that impede antibiotics and immune cells, thus providing a shielded environment for bacterial growth. Due to biofilm formation, some microbes can show up to 1000 fold increased resistance towards the antibiotics than the normal planktonic forms. The study was conducted to screen the crude extracts of medicinal plants used in Nepal for their in vitro antibiofilm activities.

**Methods:**

Total phenolic and total flavonoid contents were determined by using a Folin-Ciocalteau reagent and aluminium trichloride method, respectively. Resazurin assay was used to determine the minimum inhibitory concentration (MIC) and minimum bactericidal concentration (MBC). The initial antibiofilm activities and their inhibitory concentration (IC_50_) values were determined by the microtiter based modified crystal violet staining method.

**Results:**

Out of 25 different plant extracts were used for the study, methanolic extracts of 20 plants showed a biofilm inhibition activity against five different strong biofilm producing *Escherichia coli* strains. *Calotropis gigantea* exhibited inhibition against all five different *E. coli* strains with IC_50_ values ranging from 299.7 ± 20.5 to 427.4 ± 2.7 μg/mL. Apart from that, *Eclipta prostrata* also showed biofilm formation inhibition, followed by *Eupatorium adenophorum*, *Moringa oleifera*, *Ocimum tenuifolium, Oxalis lantifolia, Prunus persica,* and *Urtica parviflora*. The extracts of *C. gigantea, E. prostrata, Mangifera indica, O. tenuifolium, P. persica,* and *U. parviflora* exhibited a moderate to poor MIC value ranging from 625 to 2500 μg/mL. The highest amount of phenolic content (TPC) was found in *Acacia catechu* followed by *Morus alba*, which was 38.9 and 25.1 mg gallic acid equivalents, respectively. The highest amount of flavonoid content was found in *A. catechu* followed by *M. indica*, which was 27.1 and 20.8 mg quercetin equivalents, respectively.

**Conclusion:**

Extracts of *C. gigantea*, *E. prostrata, P. persica*, *U. parviflora,* and *O. tenuifolium* showed antibacterial as well as antibiofilm activity against pathogenic and strong biofilm producing *E. coli*. Thus, extracts or the pure compound from these medicinal plants could be used as antibiotics in the future.

## Background

Antibiotics are compounds that either stop bacteria from multiplying (bacteriostatic agents) or kill them entirely (bactericidal agents). These compounds effectively reduce or eliminate bacterial populations by blocking critical bacterial cellular processes [1]. However, the antimicrobials in today’s world hit a critical level and are a global concern due to the resistance mechanism exhibited by microorganisms [2].

Bacteria that show resistance to different types of antibiotics are now a severe problem with medical interventions resulting in prolonged hospitalization and recurrent infections [3]. Similarly, biofilm formation is one of the mechanisms by which bacteria possess tolerance towards drugs [4]. The biofilm in bacteria smartly creates both physical and chemical barriers so that these antimicrobials unable to penetrate the bacterial cell. For example, ciprofloxacin binds to specific components of biofilm of *P. aeruginosa* and cannot enter the cell. Thus, the biofilm acts as a barrier to the ciprofloxacin; an antibacterial agent [5]. Bacteria gain the ability to resist antibiotics, chlorine bleach, glutaraldehyde, and other chemical disinfectants due to biofilms [6]. Furthermore, biofilm help to resist various factors like pH, nutrients scarcity, osmolarity, mechanical, and shear forces [7–9]. It also restricts antibiotics and frustrates the host’s immune cells [10, 11]. Therefore, biofilm provides higher resistance as compared to only antibiotic resistance [12]. Biofilm may make a favorable environment for horizontal gene transfer with high cell density, accumulation of genetic elements, increased genetic competence, and uptake of resistance genes [8, 13]. Due to these various reasons, there is a positive correlation between biofilm production and extended-spectrum beta-lactamase (ESBL) production that confers resistance to antibiotics [14].

Nosocomial and healthcare-associated infections are one of the leading causes of death in the USA [15]. Biofilm production by bacteria is related to about 65% of nosocomial infections and 80% of other infectious diseases [15, 16]. The manifestation of biofilm-associated infection progress from an acute to chronic illness, and may persist for an extended period [17]. Biofilm formation and antibiotic resistance by microorganisms have prompted researchers to search for new drug candidates [18].. There is less effective or no ideal biofilm inhibitor available, and the search for new ones is on demand. Several bioactive compounds and herbal medicines are derived from plant sources. Therefore, the discovery of ideal biofilm disruptors can be based on plant origin [19–24]. This study aimed to investigate the antibacterial as well as antibiofilm activity along with phytochemical screening from methanolic extracts of different selected medicinal plants from Nepal using in vitro assays.

## Methods

### Chemicals and materials

Dimethyl sulfoxide (DMSO) and methanol were purchased from Fisher Scientific. Gallic acid, quercetin, ciprofloxacin, and resazurin were purchased from Sigma-Aldrich. Muller Hinton Broth (MHB), Muller Hinton Agar (MHA), Tryptone Soy Broth (TSB), and crystal violet (CV) were purchased from Hi-media.

### Bacterial strains

Five different uropathogenic clinical *E. coli str*ains (EC1-EC5) were used, which were found to be strong biofilm producers. Their biofilm-forming capabilities, antibacterial susceptibility testing pattern and molecular identification were confirmed in our previous study [14].

### Collection of plant materials and storage

All plants were collected based on the traditional medicinal uses and ethnomedicinal knowledge of ethnic people from different parts of Nepal. All plant materials were identified by professional taxonomists at National Herbarium and Plant Laboratories, Godawari, Lalitpur, Nepal. The voucher specimens were deposited in the Department of Botany, National College, Khusibu, Kathmandu, and mentioned in Table 1. The collected plant materials were shade dried at room temperature before pulverization.

**Table 1:** Plants used for the study along with their site of collection and voucher code, plant parts used for the study, and percentage yield (%)

S.N	Voucher Code	Botanical name	Family	Common name (Nepali)	Parts used for the study	Percentage yields (%)
1	NCDB152	*Acacia catechu* (L.F.) Willd.	Fabaceae	Khayar	Leaves	23.1
2	NCDB143	*Aegle marmelos* (L.) Correa	Rutaceae	Bel	Leaves	5.5
3	NCDB151	*Artocarpus heterophyllus* Lam.	Moraceae	Katahar	Leaves	21.7
4	NCDB161	*Artemisia dubia Wall. Ex DC.*	Asteraceae	Tetipati	Leaves	12.2
5	NCDB154	*Azadirachta indica* A. Juss	Meliaceae	Neem	Leaves	10.3
6	NCDB156	*Boerhavia diffusa L.*	Nyctaginaceae	Punarnava	Leaves	8.1
7	NCDB138	*Calotropis gigantea* (L.) Dryand.	Asclepiadaceae	Aakh	Leaves	6.5
8	NCDB146	*Chrysanthemum indicum* L.	Asteraceae	Godawari	Leaves and flower (Twig)	13.4
9	NCDB150	*Cinnamomum camphora (*L.) J. Presl	Lauraceae	Kapur	Leaves	12.1
10	NCDB160	*Cinnamomum tamala (*Buch.-Ham.) T. Nees and Eberm	Lauraceae	Tejpat	Leaves and Stem (Twig)	8.4
11	NCDB144	*Eclipta prostrata* L.	Asteraceae	Bharigraj	Whole Plant	6.5
12	NCDB142	*Eupatorium adenophorum* Spreng.	Asteraceae	Banmara	Leaves	10.4
13	NCDB141	*Hypericum uralum* Buch.-Ham.ex D. Don	Hyperiacaceae	Arelu	Leaves and Flower (Twig)	22.8
14	NCDB147	*Lawsonia inermis* L.	Lythraceae	Heena	Leaves	10.1
15	NCDB139	*Mangifera indica* L.	Anacardiaceae	Aap	Leaves	14.9
16	NCDB159	*Moringa oleifera* Lam.	Moringaceae	Sitalchini	Leaves	8.2
17	NCDB153	*Morus alba* L.	Moraceae	Kimbu	Bark	10.7
18	NCDB155	*Nyctanthes arbortristis* L.	Olaceae	Parijat	Leaves and flower (Twig)	6.4
19	NCDB149	*Ocimum tenuifolium L.*	Lamiaceae	KaloTulsi	Leaves and seeds	9.8
20	NCDB145	*Oxalis lantifolia* Kunth.	Oxalidaceae	Chariamilo	Leaves and stem (Twig)	7.1
21	NCDB148	*Pistia stratiotes* L.	Araceae	Jalchobi	Leaves	9.2
22	NCDB140	*Prunus persica* (L.) Batsch	Rosaceae	Aaru	Leaves	9.3
23	NCDB157	*Shorea robusta* Gaertn.	Dipterocapaceae	Sal	Leaves	24.1
24	NCDB158	*Urtica parviflora* Robx.	Urticaceae	Sisnoo	Leaves and stem (Twig)	5.8
25	NCDB137	*Zingiber officinale* Roscoe	Zingiberaceae	Aaduwa	Leaves	10.1

### Extraction

For the extraction, the plant materials were firstly ground into a fine powder using a grinding machine (Model 404, Wayal Industries, India). The extraction was done by the cold percolation method [25]. The powder of different plants was soaked in methanol for 24 h at room temperature for three successive days. Each day, the dissolved extracts were filtered through Whatman filter paper (No. 1), collected, and then evaporated at reduced pressure below 50 °C using a rotary evaporator (Biobase Re-2010, China). The working solution was prepared in 50% DMSO. The plant extracts stock solutions were maintained at 4 °C in the refrigerator until use [26]. The percentage of yield was calculated by the following formula:

$$ \mathrm{Percentage}\ \mathrm{of}\ \mathrm{yield}\ \left(\%\right)=\left(\frac{\ \mathrm{Dry}\ \mathrm{weight}\ \mathrm{of}\ \mathrm{extract}}{\ \mathrm{Dry}\ \mathrm{weight}\ \mathrm{of}\ \mathrm{a}\ \mathrm{plant}}\right)\times 100 $$

### Qualitative phytochemical screening

All the plant extracts were diluted into 10 mg/mL from the stock solution in 50% DMSO using clean test tubes [27]. Screening of steroids, alkaloids, glycosides, tannins, flavonoids, terpenoids, and phenols was done as described in previous protocols [28–31].

### Determination of total phenolic content (TPC)

The total phenolic content of the extracts was determined using the Folin-Ciocalteu reagent, as previously described with slight modification [32, 33]. In brief, 20 μL of different concentration of the standard (10–80 μg/mL, gallic acid) and 20 μL of plant extract (500 μg/mL) was added separately with 100 μL of Folin-Ciocalteu (1:10 v/v diluted with distilled water) followed by 80 μL Na_2_CO_3_ (1 M) in each well. Then, the plate was left in the dark for 30 min, and absorbance was measured at 765 nm with a spectrophotometer (Epoch2, BioTek, Instruments, Inc., USA). Gallic acid (10–80 μg/mL) was used for constructing the standard curve.

### Determination of total flavonoid content (TFC)

The total flavonoid content (TFC) of plant extracts was determined by the colorimetric method with certain modifications [34]. Shortly, 130 μL of different concentrations of the standard (10–80 μg/mL quercetin) and 20 μL of plant extracts (500 μg/mL) with 110 μL of distilled water was added separately with 60 μL ethanol, 5 μL aluminum trichloride (AlCl_3_, 10%) and 5 μL potassium acetate (1 M) in each well. It was then left in the dark for 30 min, and absorbance was recorded at 415 nm with a UV-visible spectrophotometer (Epoch2, BioTek, Instruments, Inc., USA). Quercetin (10–80 μg/mL) was used for constructing the standard curve.

### Determination of minimum inhibitory concentration (MIC) and minimum bactericidal concentrations (MBC)

The MIC was determined by adapting a previously described protocol [35–37] with some modifications. Firstly, 100 μL of MHB was added to each well of a sterile microtiter plate. In column 1, 100 μL plant extracts were added, and serially diluted down the column by two-fold till row H and finally, 100 μL from the last well was removed to maintain the final volume of each well to 100 μL. Then, 100 μL of ciprofloxacin (0.4 mg/mL) was added, and then serially diluted up to 0.0031 mg/mL by two-fold in another column as a positive control. The bacterial culture media only were added as the control. The bacterial suspension culture was maintained at a final concentration of 10^6^ CFU/mL by diluting 1:100 the 0.5 McFarland turbidity culture in MHB. Finally, 2 μL of bacteria was added to each well except in the negative control well.

Then, the plates were incubated for 24 h at 37 °C and 30 μL resazurin (0.002%) was added to each well and further incubated for 4 h, and the plates were examined for color change. Those wells having a purple color indicated dead cells or no viable bacteria, while the pink color indicated the viable cells and MIC value noted. The MBC was determined by streaking the content of wells onto MHA plates with incubation of over 18 h at 37 °C.

### Biofilm formation inhibition assay and the determination of inhibitory concentration (IC_50_) value

The biofilm formation inhibition capabilities were evaluated according to the previously described protocol [27, 38, 39] with slight modifications. *Escherichia coli* was cultured on TSB and incubated at 37 °C till the culture turbidity matched (0.5 McFarland), and diluted on fresh and sterile TSB as 1:100 dilutions. Then, 100 μL of bacterial culture was added to each well of a 96-well microtiter plate and incubated for 4 h at 37 °C to allow cell attachment. Following incubation, 100 μL of each plant extracts at a final concentration of 500 μg/mL was added to each well. An equal volume of ciprofloxacin with a final concentration of 1.25 μg/mL was added as a positive control and MHB as negative control instead of plant extracts. In blank wells, 200 μL of MHB was used without a bacteria culture to ensure the sterility of the experiment. The plates were covered with a lid and incubated at 37 °C for 48 h. The concentration of plant extracts and ciprofloxacin was maintained below their MIC value.

After incubation, the cultures were decanted on a paper towel, rinsed two times with 200 μL sterile phosphate buffer saline (PBS) of pH 7.2. Then, the plates were heat-fixed by incubating at 60 °C for 1 h. Then, the plates were stained with 0.1% crystal violet (CV) solution for 20 min at room temperature. After CV staining, plates were washed three times with PBS of pH 7.2 to free the stain from the microtiter plates. Then, the plates were air-dried and de-stained with 200 μL of 95% ethanol (v/v) for about 30 min. Finally, the absorbance was taken at 590 nm.

The percentage of inhibition of biofilm formation was calculated by using the following formula.

$$ \mathrm{Percentage}\ \mathrm{of}\ \mathrm{inhibition}\ \left(\%\right)=\left(\frac{\mathrm{OD}\ \left(\mathrm{Negative}\ \mathrm{Control}\right)-\mathrm{OD}\left(\mathrm{Experimental}\right)}{\mathrm{OD}\kern0.5em \left(\mathrm{Negative}\ \mathrm{Control}\right)}\right)\times 100 $$

Finally, IC_50_ was calculated based on percentage inhibition with the different concentrations of plant extract (500–100 μg/mL) [20, 40, 41]. Each inhibition assay was performed in triplicate and done twice to check the reproducibility of the result.

### Statistical analysis

All the experiments were done in triplicate. The results are presented as mean ± standard by Microsoft Excel. The IC_50_ value was calculated using the EZ-Fit program (Perellela Scientific, Inc., Amherst, Mars, USA). One way ANOVA test was done to compare MIC/MBC and IC_50_ values of crude extract and the positive control (ciprofloxacin) using the Statistical Package for the Social Sciences (SPSS) version 19.0 software (IBM), and *P* values < 0.05 were considered significant.

## Results

### Plants extract yield

The yield percentage of plant extracts varies from the highest of *S. robusta* (24%) followed by *A. catechu* (23%) and *H. uralum* (22.8%). While *A. marmelos* (5.5%) extract exhibited the lowest percentage yield. All the data on the percentage of yield and parts used for the study are mentioned in Table 1.

### Phytochemical screening

Phytochemical screening revealed the presence of different phytochemical components such as steroids, terpenoids, flavonoids, tannins, phenols, glycosides, and alkaloids. The glycosides, flavonoids, phenols, and steroids were found to be present in all of the plants screened for the test (Table 2).

**Table 2:** Qualitative phytochemical screening of medicinal plants used for the study

S.N	Sample Name	Steroid Test	Terpenoid Test	Flavonoid Test	Tannin test	Phenol Test	Glycosides Test	Alkaloid Test
1	*A. catechu*	+	**+**	**+**	**+**	**+**	**+**	**+**
2	*A. marmelos*	+	**+**	**+**	**+**	**+**	**+**	–
3	*A. heterophyllus*	+	**+**	**+**	**+**	**+**	**+**	–
4	*A. dubia*	+	**+**	**+**	**+**	**+**	**+**	+
5	*A. indica*	+	**+**	**+**	**+**	**+**	**+**	–
6	*B. diffusa*	+	**+**	**+**	–	**+**	**+**	–
7	*C. gigantea*	+	–	**+**	**+**	+	**+**	**+**
8	*C. indicum*	+	**+**	**+**	–	**+**	**+**	–
9	*C. tamala*	+	**+**	**+**	**+**	**+**	**+**	**+**
10	*C. camphora*	+	**+**	**+**	**+**	**+**	**+**	+
11	*E. prostrata*	+	–	**+**	**+**	**+**	**+**	–
12	*E. adenophorum*	+	–	**+**	**+**	**+**	**+**	**+**
13	*H. uralum*	+	–	**+**	–	**+**	**+**	–
14	*L. inermis*	+	–	**+**	**+**	**+**	**+**	–
15	*M. indica*	+	**+**	**+**	**+**	**+**	**+**	–
16	*M. oleifera*	+	**+**	**+**	**+**	**+**	**+**	–
17	*M. alba*	+	**+**	**+**	**+**	**+**	**+**	–
18	*N. arbortristis*	+	**+**	**+**	**+**	**+**	**+**	–
19	*O. tenuifolium*	+	**+**	**+**	–	**+**	**+**	–
20	*O. lantifolia*	+	**+**	**+**	**+**	**+**	**+**	+
21	*P. stratiotes*	+	**+**	**+**	**+**	**+**	**+**	–
22	*P. persica*	+	**+**	**+**	**+**	**+**	**+**	+
23	*S. robusta*	+	**+**	**+**	**+**	**+**	**+**	**+**
24	*U. parviflora*	+	–	**+**	**+**	**+**	**+**	–
25	*Z. officinale*	+	**+**	**+**	**+**	**+**	**+**	**+**

A positive sign (+) indicates the presence of that bioactive compound while negative sign (-) indicates the absence of that compound

### Total phenolic content (TPC) and total flavonoid content (TFC)

Methanolic extract of *A. catechu* showed the highest TPC value of 38.9 ± 0.09 mg GAE/gm and extract of *M. oleifera* showed the lowest TPC value of 0.4 ± 0.01 mg GAE/gm. The extract of *A. catechu* showed the highest TFC value of 27.1 ± 0.12 mg QE/gm and the extract of *C. camphora* showed the lowest TFC value of 1.1 ± 0.04 mg QE/gm (Table 3).

**Table 3:** The semi-quantitative detection of TPC and TFC of medicinal plants used for the study

S.N.	Plants used for the study	Total phenolic content (TPC) (TPC ± SEM)	Total flavonoid content (TFC) (TFC ± SEM)
		(mg GAE/gm)^**a**^	(mg QE/gm)^**b**^
1	*A. catechu*	38.9 ± 0.09	27.1 ± 0.12
2	*A. marmelos*	5.5 ± 0.02	2.8 ± 0.06
3	*A. heterophyllus*	18.9 ± 0.03	1.4 ± 0.03
4	*A. dubia*	5.8 ± 0.05	2.9 ± 0.04
5	*A. indica*	3.3 ± 0.01	9.2 ± 0.01
6	*B. diffusa*	5.9 ± 0.01	8.8 ± 0.01
7	*C. gigantea*	6.7 ± 0.02	4.0 ± 0.07
8	*C. indicum*	9.7 ± 0.02	10.1 ± 0.02
9	*C. tamala*	17.3 ± 0.02	4.5 ± 0.07
10	*C. camphora*	1.0 ± 0.01	1.0 ± 0.04
11	*E. prostrata*	4.2 ± 0.07	2.9 ± 0.04
12	*E. adenophorum*	6.0 ± 0.01	8.3 ± 0.04
13	*H. uralum*	16.9 ± 0.05	18.2 ± 0.05
14	*L. inermis*	7.8 ± 0.01	7.2 ± 0.06
15	*M. indica*	21.5 ± 0.06	20.8 ± 0.06
16	*M. oleifera*	0.4 ± 0.01	1.6 ± 0.01
17	*M. alba*	25.1 ± 0.07	5.3 ± 0.21
18	*N. arbortristis*	1.4 ± 0.21	2.9 ± 0.01
19	*O. tenuifolium*	3.2 ± 0.01	2.0 ± 0.01
20	*O. lantifolia*	6.1 ± 0.01	4.9 ± 0.04
21	*P. stratiotes*	1.1 ± 0.02	2.1 ± 0.04
22	*P. persica*	0.5 ± 0.05	1.2 ± 0.01
23	*S. robusta*	18.4 ± 0.02	2.9 ± 0.08
24	*U. parviflora*	3.1 ± 0.01	1.7 ± 0.03
25	*Z. officinale*	7.1 ± 0.02	4.1 ± 0.03

*SEM* Standard error of the mean

^a^The TPC values were expressed in mg gallic acid equivalent per gram, and ^b^TFC values are expressed in mg quercetin equivalent per gram

### Antibacterial activity (MIC and MBC)

The extracts of *C. gigantea, E. prostrata, M. indica, O. tenuifolium, P. persica,* and *U. parviflora* exhibited a moderate to poor MIC value ranging from 0.625 mg/mL to 2.5 mg/mL and presented in Table 4.

**Table 4:** Minimum inhibitory concentration (MIC) and Minimum bactericidal concentration (MBC) of plant extract against *E. coli* test strains

S.N.	Plant Extracts	Bacteria used for the study and concentration of tested extract (mg/mL)										***P*** value (ANOVA)
		EC1^**a**^		EC2^**a**^		EC3^**a**^		EC4^**a**^		EC5^**a**^		
		MIC	MBC	MIC	MBC	MIC	MBC	MIC	MBC	MIC	MBC	
1	*A. catechu*	5	10	10	–	5	10	5	10	5	10	*P* = 0.001
2	*A. marmelos*	10	–	1.25	5	5	10	5	10	1.25	1.25	
3	*A. heterophyllus*	5	10	10	–	10	–	5	10	10	–	
4	*A. dubia*	2.5	5	2.5	5	5	10	2.5	5	2.5	2.5	
5	*A. indica*	5	10	10	–	–	–	10	–	10	–	
6	*B. diffusa*	2.5	5	2.5	5	5	10	5	10	2.5	5	
7	*C. gigantea*	1.25	2.5	1.25	2.5	2.5	2.5	2.5	2.5	2.5	2.5	
8	*C. indicum*	5	5	2.5	5	1.25	1.25	2.5	5	5	5	
9	*C. tamala*	5	5	2.5	5	1.25	1.25	2.5	5	2.5	5	
10	*C. camphora*	5	10	5	10	5	10	5	10	10	10	
11	*E. prostrata*	2.5	5	5	10	2.5	5	2.5	2.5	1.25	2.5	
12	*E. adenophorum*	5	10	5	10	10	–	5	10	10	–	
13	*H. uralum*	5	5	5	10	5	10	–	–	2.5	5	
14	*L. inermis*	–	–	–	–	–	–	–	–	–	–	
15	*M. indica*	1.25	5	2.5	2.5	1.25	5	2.5	2.5	1.25	2.5	
16	*M. oleifera*	2.5	5	5	10	1.25	5	5	10	2.5	2.5	
17	*M. alba*	5	10	5	10	0.625	2.5	2.5	5	5	5	
18	*N. arbortristis*	5	5	2.5	2.5	2.5	2.5	2.5	5	2.5	5	
19	*O. tenuifolium*	1.25	2.5	2.5	5	2.5	5	2.5	2.5	2.5	5	
20	*O. lantifolia*	2.5	5	5	10	2.5	2.5	2.5	2.5	1.25	2.5	
21	*P. stratiotes*	2.5	5	5	10	5	10	2.5	2.5	5	10	
22	*P. persica*	2.5	5	2.5	5	2.5	5	2.5	2.5	5	5	
23	*S. robusta*	–	–	10	–	–	–	–	–	–	–	
24	*U. parviflora*	1.25	2.5	1.25	2.5	0.625	2.5	2.5	5	1.25	1.25	
25	*Z. officinale*	5	10	5	10	10	–	10	10	2.5	5	
26	Ciprofloxacin^b^	0.0125	0.025	0.0062	0.0125	0.0125	0.0125	0.0125	0.0125	0.0062	0.0062	

The MIC/MBC values of test extracts are significantly different (*P* < 0.05) from the positive control (ciprofloxacin)

^a^indicates the five different *E. coli* test strains used for the study

^b^indicates the positive control (antibiotic) used for the study

- = No MIC/MBC values were recorded at the concentration of 10 mg/mL

### Biofilm formation inhibition

The extracts of *C. gigantea, E. prostrata,* and *M. oleifera* have shown more than 60% of biofilm inhibition in most of the *E. coli* strains as presented in Table 5. *Eclipta prostata* showed 72.4% inhibition against EC1, which was the highest among methanolic extract of all plants. Meanwhile, *S. robusta* showed the lowest (25.9%) biofilm inhibition.

**Table 5:** Effect of methanol crude extracts of selected plants against biofilm formation by uropathogenic *E. coli*

S.N.	Plants	Percentage of inhibition (%) at 500 μg/mL				
		Bacteria used for the study (***E. coli;*** EC)				
		EC1^**a**^	EC2^**a**^	EC3^**a**^	EC4^**a**^	EC5^**a**^
1	*A. catechu*	44.4	66.4	50.1	38.9	58.3
2	*A. marmelos*	56.2	75.4	46. 6	60.2	73.1
3	*A. heterophyllus*	35.4	43.9	21.5	27.1	39.5
4	*A. dubia*	60.4	65.9	29.1	62.3	65.9
5	*A. indica*	52.1	62.4	45.4	43.8	49.4
6	*B. diffusa*	58.1	72.2	23.8	60.6	68.7
7	*C. gigantea*	64.2	77.1	69.8	68.2	73.2
8	*C. indicum*	47.2	70.2	61.1	62.3	65.9
9	*C. tamala*	35.4	63.4	57.9	43.1	49.7
10	*C. camphora*	41.9	67.8	35.6	49.5	56.2
11	*E. prostrata*	72.4	71.2	53.1	69.1	77.4
12	*E. adenophorum*	56.7	65.2	56.1	52.4	67.6
13	*H. uralum*	37.2	66.7	43.4	61.1	71.4
14	*L. inermis*	45.8	67.7	32.1	58.1	69.2
15	*M. indica*	59.7	76.7	60.7	52.9	68.6
16	*M. oleifera*	60.5	64.1	65.1	51.4	71.2
17	*M. alba*	44.8	65.3	61.5	48.3	52.5
18	*N. arbortristis*	37.1	74.4	24.1	67.2	70.7
19	*O. tenuifolium*	63.1	76.1	51.7	61.1	69.2
20	*O. lantifolia*	57.1	77.2	56.8	65.9	72.5
21	*P. stratiotes*	26.4	73.1	41.1	64.8	68.3
22	*P. persica*	63.1	78.4	62.1	65.9	61.6
23	*S. robusta*	26.1	60.3	29.8	39.3	49.3
24	*U. parviflora*	59.3	76.1	62.5	61.4	72.8
25	*Z. officinale*	40.6	70.4	39.4	56.4	72.1
26	Ciprofloxacin^b^	46.3	65.8	58.7	53.7	56.3

^a^indicates the five different *E. coli* test strains used for the study

^b^indicates the positive control (antibiotic) used for the study

The 8 among 25 plant extracts were selected to calculate their inhibitory concentration (IC_50_) values against respective bacterial strains based on the preliminary result of biofilm inhibition. *Nyctanthes arbortristis* (IC_50_ = 246.2 ± 22.9 μg/mL) followed by *E. prostrata* (289.5 ± 12.3 μg/mL) and *C. gigantean* (299.7 ± 20.5 μg/mL) showed a moderate biofilm inhibition against EC4 as compared to ciprofloxacin (1.9 ± 0.1 μg/mL). Similarly, *E. prostrata* (IC_50_ = 303.1 ± 16.7 μg/mL) followed by *C. gigantea* (389.8 ± 7.5 μg/mL) and *P. persica* with (445.4 ± 8.1 μg/mL) showed a moderate biofilm inhibition against EC1 as compared to ciprofloxacin (1.8 ± 0.2 μg/mL) (Table 6).

**Table 6:** Biofilm formation inhibition (IC_50_) of methanolic extracts against *E. coli* test strains

S.N.	Plants	IC_**50**_ value (μg/mL)					***P*** value (ANOVA)
		Bacteria used for the study					
		EC1^**a**^	EC2^**a**^	EC3^**a**^	EC4^**a**^	EC5^**a**^	
1	*A. marmelos*	*–*	*–*	*–*	*–*	376.2 ± 3.6	*P* = 0.001
2	*C. gigantea*	389.8 ± 7.5	359.6 ± 10.8	350.1 ± 21.5	299.7 ± 20.5	427.4 ± 2.7	
3	*E. prostrata*	303.1 ± 16.7	–	–	289.5 ± 12.3	356.1 ± 11.1	
4	*M. oleifera*	*–*	*–*	314.5 ± 16.9	*–*	*–*	
5	*N. arbortristis*	*–*	*–*	–	246.2 ± 22.9	–	
6	*O. lantifolia*	–	305.7 ± 21.9	–	–	–	
7	*P. persica*	445.4 ± 8.1	320.9 ± 20.8	–	–	–	
8	*U. parviflora*	*–*	*–*	410.5 ± 10.7	*–*	*–*	
9	Ciprofloxacin^b^	1.8 ± 0.2	0.9 ± 0.1	0.9 ± 0.1	1.9 ± 0.1	0.9 ± 0.1	

The IC_50_ values of test extracts are significantly different (*P* < 0.05) from the positive control (ciprofloxacin)

^a^five different *E. coli* strains

^b^the positive control of the test (Antibiotic used as a positive control)

- = plant extracts were not taken for their IC_50_ value determination for respective test strains

## Discussion

Plants are the foundation for many pharmaceuticals however only a small fraction of plant species have been investigated for the presence of antimicrobial compounds [42]. Methanol is the choice of solvent because polar and moderately polar compounds like terpenoids, tannins, flavones, and polyphenols can be extracted by methanol [43, 44]. The bioactivity of plant extracts varies based on the geographical source, harvest time, storage conditions, soil conditions, drying method, etc. [45, 46]. The phytoconstituents of the plant are responsible for the inhibition of biofilm, such as glycoside acts by breaking the larger polysaccharides present in EPS into smaller monomeric subunits. Alkaloids and their derivatives disrupt fimbriae and other adhesions used for cell adhesion and biofilm formation, while tannic acid inhibits quorum-sensing (QS) systems in various Gram-negative bacteria [11, 20, 40]. The capacity of antibacterial components to inhibit the initial biofilm formation holds an assurance for minimizing the surface colonization by microbes [47].

A low concentration of the extracts may be enough to prevent the biofilm attachment process, while a higher concentration may be required to disrupt preformed biofilm [48]. *Calotropis gigantea* indicated the presence of alkaloids, flavonoids, glycosides, saponins, tannins, steroids, triterpenoids, and phenols (Table 2 and Table 3). The synergistic effect of these phytoconstituents might be responsible for antibacterial as well as biofilm formation inhibition [49].

The study conducted by Suga and Smith, in 2003 found that the extracts of *E. prostrata* contain several phytochemicals like tannic acid, alkaloids, caffeic acid, which act as effective quorum sensing inhibitors [50]. Quorum-sensing, in particular, autoinducer-2 mediated cell-cell signaling, was proposed as a significant regulatory factor for biofilm production in *E. coli* [51]. Ellagitannin, a natural product from various medicinal plants, has also shown anti-quorum sensing activity against various Gram-negative bacteria, including *P. aeruginosa* [52]. Besides the effect on the QS process, many compounds isolated from plant extracts such as proanthocyanidins, licochalcone A, 1-deoxynojirimycin (DNJ), hydroxychavicol, macelignan, panduratin, 3,12-oleandione had shown biofilm inhibition [53–57]. Also, different phenolic compounds isolated from plants like gallic acid, chlorogenic acid, and quercetin inhibit quorum sensing activity, which is a major step for biofilm formation in both Gram-positive and Gram-negative microorganisms [58].

Ursolic acid, a constituent of *O. tenuifolium,* has been found to modulate the genes, *cheA, tap, tar, motAB, hslSTV*, and *mopAB*, which are responsible for chemotaxis, mobility, and heat shock response, and ultimately affects biofilm formation [59]. The motility genes, and AI-2 quorum sensing genes in Enterohemorrhagic *Escherichia coli* O157: H7 (EHEC) has been found to be affected by plant extracts [60, 61]. Therefore, the biofilm formation inhibition mechanism may be through the modulation of genes as well.

Molecules with a lower MIC value for antimicrobial, and lower IC_50_ value for antibiofilm activities could be potent antibiotic. The lowest IC_50_ value was exhibited by *N. arbortristis* among all the tested plant extracts (Table 6), and it also showed a lower MIC value against the EC4 strain (Table 4). A similar pattern was seen in *C. gigantea*. It seems there is a positive correlation between MIC and IC_50_ values, however, these data are not sufficient to conclude.

Although *A. catechu* was found to be rich in phenols, flavonoids (Table 2) and contained all the tested phytochemicals (Table 3), it was not giving the best result as an antimicrobial and biofilm inhibitor. The extract of *C. gigantea* exhibited the best result during our study and antiarol, blumenol A, mudarine, calotropin, uscharin, and calotoxin are the reported fully characterized molecules isolated from *C. gigantea* [62]. Furthermore, the major constituents of *E. prostrata* are phytosterol, beta-amyrin, polyacetylene, caffeic acid, stigmasterol, and daucosterol [63]. Similarly, the major constituents of *P. persica* are vesveratrol, silymarin, quercetin, curcumin, β-sitosterol, and prunasin [64]. The IC_50_ and the MIC/MBC values of extracts were weaker than the positive control (ciprofloxacin), and significantly different (*P* < 0.05). Crude extracts usually exhibit weaker activity than pure compounds. This may be due to the active ingredient or the molecule might have diluted with other molecules, or maybe due to the antagonistic effect of other molecules present in the extracts. Although the result of ANOVA was significant, the post hoc test was not reported because the result of crude extract was weaker than the standard pure compound in this study.

The antimicrobial and antibiofilm formation inhibition testing with those above-mentioned molecules may lead to the discovery of new antibiotics. However, further investigation for the full characterization of the molecules from these plants is suggested.

## Conclusion

The study concluded that the plant extracts exhibiting antibacterial property coupled with antibiofilm activity. Therefore, these extracts might serve as potential candidates for developing biofilm inhibitors and may act as potent drugs against antibiotic-resistance biofilm-producing bacteria.

## Data Availability

Herbaria of plant specimens and the identification information sheets are stored in the Department of Botany, National College, Khusibu, Nayabazar, Kathmandu, Nepal and can be retrieved when necessary. Data supporting this manuscript are protected in laboratory information systems at the Department of Biotechnology, National College, Khusibu, Nayabazar, Kathmandu, Nepal and are available from the corresponding author on reasonable request.

## Abbreviations

CV: Crystal Violet
DMSO: Dimethyl Sulfoxide
*E. coli,* EC: *Escherichia coli*
MBC: Minimum Bactericidal Concentrations
MIC: Minimum Inhibitory Concentration
MHA: Muller Hinton Agar
MHB: Muller Hinton Broth
TSB: Tryptone Soy Broth
QS: Quorum Sensing
TFC: Total Flavonoid Content
TPC: Total Phenolic Content
